# Ocular Findings in COVID-19 Patients: A Review of Direct Manifestations and Indirect Effects on the Eye

**DOI:** 10.1155/2020/4827304

**Published:** 2020-08-27

**Authors:** Federica Bertoli, Daniele Veritti, Carla Danese, Francesco Samassa, Valentina Sarao, Nicolò Rassu, Tommaso Gambato, Paolo Lanzetta

**Affiliations:** ^1^Department of Medicine—Ophthalmology, University of Udine, Udine, Italy; ^2^Clinica Oculistica, Azienda Sanitaria Universitaria Friuli Centrale, Udine, Italy; ^3^Scientific Institute I.R.C.C.S. “Eugenio Medea”—“La Nostra Famiglia”, Udine, Italy; ^4^Istituto Europeo di Microchirurgia Oculare (IEMO), Udine, Italy

## Abstract

The novel pandemic coronavirus disease 2019 (COVID-19), caused by the severe acute respiratory syndrome coronavirus 2 (SARS-CoV-2), has challenged the medical community. While diagnostic and therapeutic efforts have been focused on respiratory complications of the disease, several ocular implications have also emerged. SARS-CoV-2 RNA has been found in tears of the infected patients, and reports suggest that the ocular surface could serve as a portal of entry and a reservoir for viral transmission. Clinically, COVID-19 has been associated with mild conjunctivitis, which can be the first and only symptom of the disease. Subtle retinal changes like hyperreflective lesions in the inner layers on optical coherence tomography (OCT), cotton-wool spots, and microhemorrhages have also been reported. In addition, COVID-19 has been associated with an increased incidence of systemic diseases like diabetes mellitus and Kawasaki disease, which are particularly relevant for ophthalmologists due to their potentially severe ocular manifestations. Several treatment strategies are currently under investigation for COVID-19, but none of them have been proved to be safe and effective to date. Intensive care unit patients, due to risk factors like invasive mechanical ventilation, prone position, and multiresistant bacterial exposure, may develop ocular complications like ocular surface disorders, secondary infections, and less frequently acute ischemic optic neuropathy and intraocular pressure elevation. Among the array of drugs that have shown positive results, the use of hydroxychloroquine and chloroquine has raised a concern due to their well-known retinal toxic effects. However, the risk of retinal toxicity with short-term high-dose use of antimalarials is still unknown. Ocular side effects have also been reported with other investigational drugs like lopinavir-ritonavir, interferons, and interleukin-1 and interleukin-6 inhibitors. The aim of this review was to summarize ophthalmological implications of SARS-CoV-2 infection to serve as a reference for eye care and other physicians for prompt diagnosis and management.

## 1. Introduction

Severe acute respiratory syndrome coronavirus 2 (SARS-CoV-2) has led to a global epidemic with more than 4 million confirmed cases and 280,000 deaths worldwide thus far. The disease caused by SARS-CoV-2 has been named “COVID-19” (where “CO” stands for corona, “VI” for virus, “D” for disease, and “19” indicates the year in which it occurred).

During December 2019, several cases of pneumonia of unknown origin were reported in Wuhan, the capital of Hubei Province in China [1]. A young ophthalmologist, Dr. Li Wenliang, was the first physician to report similarities with severe acute respiratory syndrome (SARS). Dr. Wenliang himself contracted the virus after treating an infected glaucoma patient and subsequently passed away [2].

A novel RNA betacoronavirus was identified as the causative pathogen. The phylogenetic analysis suggests that bats may be the original host of the virus. The first infected people were exposed to live animals being sold in a wet market in Wuhan. Transmission of the disease from human to human mainly occurs via direct contact or droplets from an infected patient through coughing or sneezing [3]. Coronaviruses (CoVs) affect a wide range of birds and mammals. Their ability to undergo mutations facilitates the transmission from animals to humans. Beyond SARS-CoV-2, two human CoVs previously emerged as capable of causing respiratory failure: SARS-CoV and Middle East Respiratory Syndrome (MERS)-CoV. There are no reported ocular manifestations associated with SARS-CoV infection. Only one case report describes SARS-CoV positivity of a tear sample analyzed via polymerase chain reaction (PCR), while other testing methods resulted negative. As far as MERS-CoV is concerned, there is no evidence of either ocular manifestations or viral load in tear samples [4]. CoVs are single-stranded positive-sense RNA viruses. The genome codes for both structural and nonstructural proteins. Structural proteins permit the viral infection and replication. Specifically, the surface spike glycoprotein (S-protein) enables the attachment between CoV and host cells [5]. There is a structural similarity between the receptor-binding domain of SARS-CoV and SARS-CoV-2. The lung epithelial cells are their primary target. They bind to the same primary cellular receptor, which is human angiotensin-converting enzyme 2 (ACE-2), causing potentially severe infections in both the upper and lower respiratory tracts [3]. The clinical manifestations are variable. Patients with mild symptoms usually recover quickly, while severe cases may develop progressive respiratory failure, potentially leading to death [5]. Currently, reverse transcriptase-polymerase chain reaction PCR (RT-PCR detection of the viral genome in the upper respiratory tract swabs is the most reliable diagnostic test [6]. At present, neither a vaccine nor a specific antiviral treatment is available. The aim of this review is to sum up the ophthalmological features of the COVID-19 and the effects that its therapies may have on the ocular tissues.

## 2. Methods

A thorough literature search was conducted in the PubMed database (https://pubmed.ncbi.nlm.nih.gov/) using as keywords “COVID-19” or “SARS-CoV-2” combined with “eye” or “ophthalmology.” In addition, other appropriate keywords were used depending on the article section (e.g., “COVID-19” AND “Kawasaki,” “Kawasaki” AND “eye,” “eye” AND “intensive care,” and “eye” AND “chloroquine”). Considering the peculiarity of the situation and the rapidly growing body of the literature, a considerable part of the articles included are of low quality (case reports, letters, and editorials). Moreover, for the same reasons, non-peer-reviewed articles have also been included, when appropriate.

## 3. Results and Discussion

### 3.1. Eye Complications during the Course of COVID-19

#### 3.1.1. Conjunctivitis

Since SARS-CoV-2 may lead to respiratory failure, most of the diagnostic and therapeutic efforts are focused on the consequences of the infection in the respiratory tract. Nevertheless, it is important to be aware that other manifestations of the disease exist, especially because they are linked to alternative ways of transmission. During the COVID-19 outbreak, conjunctivitis was reported as a manifestation of the disease and viral RNA was found in the patients' tears.

The first reported case of SARS-CoV-2 conjunctivitis affected a member of the Chinese panel for pneumonia, who developed conjunctivitis and COVID-19 after performing an inspection in the Wuhan Fever Clinic without wearing eye protection. This case highlights the potential conjunctival transmission route [7].

The exact pathogenetic mechanisms of the conjunctival infection are still unknown. The ocular surface could potentially serve as a portal of entry through exposure to aerosolized droplets or hand-eye contact. ACE-2 receptor, together with cell surface protease enzyme (TMPRSS2), is the key factor that is responsible for binding with the virus and allows access of the virus into the host cell. The presence of these receptors on the ocular surface is controversial. A study showed, by the means of immunohistochemical analysis, that there is a distinct presence of the ACE-2 receptor on the conjunctiva, limbus, and cornea. Conjunctival specimens also express TMPRSS2 [8].

Other authors did not find evidence of significant conjunctival ACE-2 expression [9]. A recent study demonstrated that consistent expression of TMPRSS2 cannot be found in conjunctival samples, while it is present in some pterygium samples [10]. It has been hypothesized that the direct viral insult is the basis of the systemic infection, which is subsequently sustained by a severe immune reaction leading to potentially massive tissue damage. It is also likely that the entity of the immune response is not equal in all the patients, due to variants in the inflammasome genes. The subsequent life-threatening hyperinflammatory syndrome is known as macrophage activation syndrome. Therefore, both autoinflammatory and autoimmune response may be involved. Since it is renowned that some forms of conjunctivitis are sustained by an autoimmune mechanism, it may be possible that macrophage activation syndrome plays a role also in the pathogenesis of SARS-CoV-2 conjunctivitis [4].

It is likely that SARS-CoV-2 has a low conjunctival replication. Still, the inoculation of SARS-CoV-2 may nevertheless happen via infected tears which transport the virus through the nasolacrimal duct towards the nasopharynx. Also, it is possible that the virus infects the conjunctiva through still unidentified receptors. Sustained replication in conjunctiva is suggested by a case report of a patient with persistent positivity of the conjunctival swab, despite nasopharyngeal tests remaining negative [11].

The signs of COVID-19 conjunctivitis are similar to the presentation of other viral forms. The patients usually present mostly bilateral conjunctival hyperemia, chemosis, follicular reaction of the tarsal conjunctiva, epiphora, watery discharge, mild eyelid edema, and enlarged preauricular and submaxillary lymph nodes. At present, there are no reports of blurred vision or sight-threatening events [12]. In the literature, there is only one case report that noted a patient with monolateral keratoconjunctivitis as the first manifestation of COVID-19 [13], while another study described an unusual bilateral pseudomembranous conjunctivitis in an intubated patient successfully treated with azithromycin eye drops twice a day, low doses of dexamethasone, and mechanical debridement [14].

The exact incidence of conjunctivitis in COVID-19 patients is still unclear, ranging between 0.8% and 31.6% [1, 12, 15].

In patients affected by conjunctivitis, a conjunctival swab is usually obtained and analyzed via RT-PCR. However, there is a low percentage of positive results, confirmed by different studies. It is possible that, in negative cases, the viral load is inferior to the threshold of test detection. Also, some patients had already started systemic antiviral therapy before the swab. It is possible that there is a low chance of transmission through tears [15]. Interestingly, a study revealed that patients with ocular symptoms from COVID-19, compared with patients with no ocular manifestations, had higher white blood cells and neutrophil counts and higher levels of procalcitonin, C-reactive protein, and lactate dehydrogenase [12]. A meta-analysis concluded that conjunctivitis in COVID-19 patients is usually associated with a more severe form of the disease and a worse outcome [16].

There are reports suggesting that conjunctivitis may be the first manifestation of the disease, followed by the onset of systemic symptoms after a variable amount of time [17]. However, the possibility that conjunctivitis may be the only manifestation of the disease must also be taken into account [18]. Interestingly, there are reports of SARS-CoV-2 RNA being isolated on the normal conjunctiva of COVID-19 patients without ocular manifestations. This may imply a viral spreading via conjunctival contact, even in patients without conjunctivitis [19]. The conjunctival manifestations of COVID-19 appear to be self-limiting. In some cases, the use of ribavirin and ganciclovir as topical therapy was followed by improvement of signs and symptoms [20].

Together, these results indicate that ocular surface cells are susceptible to infection by SARS-CoV-2 and could, therefore, serve as a portal of entry, as well as a reservoir for person-to-person transmission of this virus. Therefore, it is important to adopt safety practices to prevent infection and virus spread and to assume an extra caution behavior in ophthalmology [21, 22].

#### 3.1.2. Kawasaki Disease

Kawasaki disease (KD) is an acute and usually self-limiting vasculitis of the medium caliber vessels, which mostly exclusively affects young children, and it is characterized by fever, oropharyngeal and extremity changes, polymorphous rash, and unilateral cervical lymphadenopathy. The cause of KD remains unknown, despite several decades of investigation. However, earlier evidence suggests that an infectious agent may trigger a cascade that causes the illness.

The Bergamo province in Italy, which was extensively affected by SARS-CoV-2 epidemic, observed a strong association between an outbreak of Kawasaki-like disease and COVID-19. Specifically, some authors reported a 30-fold increased incidence of a severe form of KD with a percentage of 80% of children positive for COVID-19 serology [23].

Other studies and news media also report unprecedented clusters of patients affected by KD in the UK (up to 100 cases, but none tested positive for COVID-19) and New York state (over 1,000 new cases) [24, 25].

The first described case of KD with concurrent COVID-19 infection was observed in a 6-month-year-old female in the setting of fever and minimal respiratory symptoms. The baby, tested positive for COVID-19, had limbic sparing conjunctivitis, prominent tongue papilla, a blanching, polymorphous, maculopapular rash, and swelling of the hands and lower extremities [25].

The KD has particular relevance for ophthalmologists due to its potential ocular involvement. Most frequent ocular manifestations are iridocyclitis, punctate keratitis, vitreous opacities, papilledema, subconjunctival hemorrhage, and conjunctival injection. The latter is usually bilateral, painless, nonexudative, and limbic sparing [26].

As the SARS-CoV-2 epidemic evolves with time, a similar outbreak of Kawasaki-like disease is expected in countries around the world. Ophthalmologists should, therefore, be aware of the potential ocular manifestations and consider appropriate treatment if needed.

#### 3.1.3. Diabetic Retinopathy

Due to the global coronavirus outbreak, many countries worldwide have adopted isolation policies in order to assure social distancing. Physical inactivity and sedentary behavior imposed by lockdown policies may be deleterious for patients. Daily step reduction from 10,000 to 1,500 steps in healthy adults can lead to impaired insulin sensitivity and slower lipid metabolism, increasing visceral fat and decreasing lean body mass and worsening cardiovascular performances. This may have unforeseen consequences on public health, such as new onset or worsening of diabetes mellitus, leading to increased referrals to ophthalmologists for eye complications related to diabetes [27].

Future epidemiological studies may reveal a possible lockdown implication in increased incidence of severe diabetic retinopathy cases during COVID-19 pandemic.

#### 3.1.4. Retinal Findings

A recent report analyzing optical coherence tomography (OCT) findings in 12 patients tested positive for SARS-CoV-2 infection showed hyperreflective lesions at the level of the ganglion cell and inner plexiform layers on OCT. This bilateral finding was present in all patients and was more prominent at the papillomacular bundle. Results of OCT angiography and ganglion cell complex analysis appeared normal. Four patients (33%) presented subtle cotton-wool spots and microhemorrhages along the retinal arcade on fundus photography. No signs of intraocular inflammation, visual acuity alteration, or abnormal pupillary reflexes were detected [28]. Recently, concerns have been raised regarding the possible misinterpretation of these findings, suggesting that the hyperreflective areas may simply represent normal retinal vessels [29]. A recent paper by Zhang et al. suggests that the leading factor in the pathogenesis of microcircular damage in COVID-19 patients is complement-mediated thrombotic microangiopathy (TMA) [30]. Complement system activation has been previously described as directly responsible of ocular vascular damage, with rare cases of atypical hemolytic uremic syndrome, leading to retinal artery and vein occlusions [31]. It is also worthy of consideration that high serum levels of C3 complement factor have also been linked to increased risk of developing diabetic retinopathy, nephropathy, and neuropathy, via endothelial dysfunction and thrombosis [32]. Immunohistochemical analysis conducted on the human eye has shown that the ciliary body, choroid, retina, and retinal pigment epithelium (RPE) express significative levels of ACE receptors [33]. Since COVID-19 is able to target vascular pericytes expressing ACE-2, viral infection could lead to complement-mediated endothelial cell dysfunction, microvascular damage, and thus ocular circulation involvement [34]. COVID-19-associated coagulopathy may predispose to a spectrum of thromboembolic events. Numerous cases of deep venous thrombosis, pulmonary embolism, and large-vessel ischemic strokes in patients with COVID-19 have been described. At the time of this review, only one case of isolated central retinal artery occlusion secondary to COVID-19 has been published [35], while an increase in the incidence of retinal vein occlusions has not been reported. The role of thrombophilic risk factors in the pathogenesis of retinal vein occlusions is still controversial, and some authors suggest that cardiovascular risk factors for artery diseases play a more important role than coagulation disorders [36]. Future research may disclose a possible COVID-19 implication in retinal vascular pathology and an increased incidence of retinal vascular occlusions during the COVID-19 pandemic.

#### 3.1.5. Neuro-Ophthalmological Complications

Neurological complications of COVID-19 include polyneuritis, Guillain–Barr*é* syndrome (GBS), meningitis, encephalomyelitis, and encephalopathy. Reports of patients who were diagnosed with COVID-19 after presenting with diplopia and ophthalmoparesis and abnormal perineural or cranial nerve MRI findings have been described in the literature [37]. Oculomotor nerve palsy could be triggered by direct virus invasion or inflammatory factors related to viral infection or could be secondary to neurological complications such as GBS, acute disseminated encephalomyelitis, or transverse myelitis [38]. Although animal models suggest ocular lesions could include optic neuritis, an increase in the incidence of ischemic or inflammatory optic neuropathies cases related to COVID-19 has not been reported in the literature yet [5].

### 3.2. Ocular Complications in Intensive Care Unit Patients

Prevalence of acute respiratory distress syndrome (ARDS) among COVID-19 patients has been reported to be 17% [39]. ARDS is a life-threatening condition, which requires respiratory support in an intensive care unit (ICU). A recently published study on 1,591 COVID-19 patients admitted to ICUs of the Lombardy Region (Italy) reports an admission rate of 9%, while other studies report even higher rates, up to 32% [40, 41]. It must be noted that those patients who need respiratory support in an ICU have high propensity to develop ocular complications. The incidence of eye-related complications in ICU patients in different studies varies from 3% to 60%. Ocular surface disorders, intraocular pressure (IOP) elevation, and anterior and posterior segment disorders are the most frequent manifestations [42].

#### 3.2.1. Ocular Surface Disorders

The most common ocular complications in ICU patients are surface disorders, which have been reported to occur in up to 60% of critically ill patients and can range from mild conjunctival irritation to severe infectious keratitis [42]. ICU patients present several risk factors for surface disorders, some of which related to the treatments, while others to the ICU environment itself, e.g., exposure to many potentially multiresistant bacteria [43]. In mechanically ventilated patients, the main ocular surface defense mechanisms are impaired. Muscle relaxants and sedating agents reduce the tonic contraction of the orbicularis oculi, thus leading to lagophthalmos. Moreover, they inhibit the blink reflex and Bell's phenomenon and reduce tear production [44]. As a result, an exposure keratopathy of variable severity may develop. Continuous positive airway pressure (CPAP) and oxygen masks have a drying effect on the ocular surface. Exposure keratopathy affects up to 42% of ICU patients and 60% of those sedated for more than 48 hours [45]. It has been reported that ill-fitting Venturi masks can cause corneal abrasions by rubbing on the eye [46]. In addition to the direct damage, exposure keratopathy can also lead to secondary infections, such as conjunctivitis and keratitis.

Conjunctival chemosis is commonly seen in ICU patients and, when particularly severe, may contribute to lagophthalmos and reduced ocular surface lubrication. Risk factors for developing conjunctival chemosis include reduced venous return from the eye (due to positive pressure ventilation or tight endotracheal tube taping) and increased hydrostatic pressure (mainly due to prolonged recumbency, especially if prone). Prone position has been shown to decrease mortality in ARDS patients, and some authors recommend it for a minimum of 12 hours per day [47, 48]. Since it increases venous pressure in the head, it can theoretically also cause subconjunctival hemorrhage, a condition usually completely benign although it may lead to surface disorders, if extensive [49, 50]. In mechanically ventilated patients, the positive end-expiratory pressure may lead as well to subconjunctival hemorrhage because of an increase in intrathoracic pressure and consequently in central venous pressure [51].

Data on the incidence of infectious keratitis and conjunctivitis in mechanically ventilated patients are not available. However, a study on 134 patients without preexisting ocular surface disorders who underwent sedation and respiratory support reports that 77% of patients were colonized by at least one bacterial species other than normal flora and 40% by multiple species [43]. The most common isolates were *Pseudomonas aeruginosa*, *Acinetobacter* spp., and *Staphylococcus epidermidis*.

#### 3.2.2. Rare Ocular Complications

It has been reported that prone position ventilation may rarely lead to acute ischemic optic neuropathy, which causes permanent vision loss [52]. Ocular perfusion depends on IOP and ocular blood flow, which in turn depends on arterial and venous pressure and on vascular resistance [48, 49, 53]. Prone position can critically reduce ocular perfusion acting on two mechanisms. On the one hand, it increases venous pressure, and on the other hand, it also increases the IOP. IOP rises with time, up to approximately 40 mmHg after 320 minutes in the prone position [54]. This condition can also be exacerbated by ill-fitting prone face positioners [55]. In addition, systemic conditions such as diabetes, arterial hypertension, and atherosclerosis may determine an increase in vascular resistance, thus further reducing ocular blood flow [50]. It follows that those patients who are more likely to be admitted to the ICU for COVID-19 because of their comorbidities are also at higher risk to suffer from ocular hypoperfusion.

Valsalva retinopathy is a condition characterized by the sudden onset of uni- or bilateral macular preretinal hemorrhages, resulting from rupture of small superficial capillaries due to an increased venous pressure [56]. It is usually associated with activities causing a sudden increase in intrathoracic or intra-abdominal pressure. It has been reported that valsalva retinopathy can also occur due to intubation or high positive end-expiratory pressure [51, 57].

A potentially sight-threatening complication in ICU patients is acute angle-closure glaucoma. In the presence of underlying risk factors, an acute angle closure can be triggered by the prone position, as well as by many local and systemic drugs, such as anticholinergics (atropine, ipratropium bromide, tricyclic antidepressants, and antihistamine), sympathomimetics (adrenaline, noradrenaline, dopamine, ephedrine, salbutamol, and terbutaline), and others (sulfonamides derivatives and topiramate) [58, 59].

Horner's syndrome has been reported as a rare complication of central venous catheterization [60]. Its frequency was 2% in a sample of 100 patients, prospectively examined. It was likely caused either by direct trauma to the sympathetic plexus or by an expanding hematoma. A small percentage of critically ill COVID-19 patients can develop typical clinical manifestations of viral sepsis. Endogenous endophthalmitis should be considered among the possible rare complications of sepsis related to COVID-19. Till date, no reports of this condition have been described in the literature [61].

In conclusion, intensive care, and especially invasive mechanical ventilation, can be associated with several ocular complications. ICU staff must be aware of them and refer to an ophthalmologist when appropriate. Sight-threatening complications are rare, but it is crucial that they are diagnosed and treated before permanent damage occurs. Ocular surface disorders, on the other hand, are extremely common, and several studies showed that the application of a proper protocol can significantly reduce their incidence [62, 63].

### 3.3. Ocular Side Effects of Drugs Used for the Treatment of COVID-19

To date, no pharmacological therapies have been approved for the treatment of COVID-19. However, several drugs are currently under investigation, such as chloroquine (CQ) and its derivative hydroxychloroquine (HCQ), antiviral drugs, and immunomodulators [64].

#### 3.3.1. Antimalarial Drugs

The antimalarial drugs CQ and HCQ are mainly used to treat malaria, amebiasis, and rheumatologic conditions, such as systemic lupus erythematosus, rheumatoid arthritis, Sjogren's syndrome, and juvenile idiopathic arthritis. Recent studies have demonstrated their activity in vitro and in animal models against SARS-CoV-2, and the FDA has approved an emergency authorization for use of these drugs for hospitalized COVID-19 patients [65, 66]. A Chinese study found that CQ abbreviated the disease course, reduced the exacerbation of pneumonia, with pulmonary imaging findings improvements, and promoted virus-negative seroconversion [67].

Among patients with COVID-19, the use of HCQ has been shown to significantly shorten the time to clinical recovery and promote the resolution of pneumonia [68]. A recent study by Gautret et al. reported that HCQ treatment in patients affected by COVID-19 was significantly associated with viral load reduction/disappearance and that its effect was reinforced by azithromycin [69]. Further studies are underway to provide a definitive answer of the value of CQ and HCQ in patients with severe COVID-19. The antiviral efficacy of CQ and HCQ seems to be explained by (1) an increase in endosomal pH that inhibits viral fusion and replication, (2) an interference with the terminal glycosylation of the ACE-2 receptor for cell entry targeted by SARS-CoV and SARS-CoV-2, and (3) an immunomodulatory activity [64]. The dosage of CQ and HCQ is different. Patients affected by COVID-19 typically received CQ at a dose of 1,000 mg daily on day 1 and then 500 mg daily for 4 or 7 days. The dosage of HCQ is 800 mg daily on day 1, followed by 400 mg daily for 4 or 7 days.

Side effects of these drugs include QTc interval prolongation, hypoglycemia, gastrointestinal disorders, anemia, extrapyramidal disorders, and ocular complications [65, 66].

The clinical picture of HCQ and CQ ocular toxicity includes whorl-like corneal intraepithelial deposits, which are usually reversible, posterior subcapsular lens opacity, ciliary body dysfunction, and a bilateral maculopathy characterized by a ring of parafoveal RPE depigmentation that initially spares the fovea. Advanced cases of CQ and HCQ maculopathy show widespread photoreceptor loss and RPE atrophy with foveal involvement and progressive loss of visual acuity. HCQ and CQ maculopathy is not reversible and can progress even after interrupting drug assumption, probably due to a gradual decompensation of retinal cells that were metabolically injured during drug exposure [70].

The most critical risk factor for the development of CQ and HCQ toxicity is excessive daily dosage. The American Academy of Ophthalmology recommendations on screening for CQ and HCQ retinopathy suggest keeping a daily dosage inferior to 2.3 mg/kg in patients receiving CQ and less than 5.0 mg/kg in those using HCQ. Therefore, most of the patients treated with CQ and HCQ for COVID-19 receive potentially retinotoxic doses. Duration of therapy is an additional critical factor. Prolonged use of HCQ at recommended doses increases the risk of ocular toxicity, rising from less than 2% after 10 years to almost 20% after 20 years [71]. However, it is also reported that high CQ and HCQ dosages can lead to retinopathy even with shorter therapy duration. Two recent studies on patients receiving 800–1,000 mg/day of HCQ showed a 25% to 40% incidence of retinopathy within 1-2 years [72, 73]. No reports of retinal toxicity under 2 weeks of CQ or HCQ administration have been described. Thus, to date, evidence suggests that high doses of these drugs can accelerate retinal toxicity over a period of weeks to years [74].

#### 3.3.2. Antiviral Drugs

The second-generation antiretroviral drugs, lopinavir and ritonavir, are widely used for the treatment of HIV, and some reports have drawn attention to the use of these drugs as a possible treatment for patients with COVID-19 infection. Ritonavir inhibits the cytochrome P450 3A4 increasing the half-life of lopinavir; therefore, these two drugs are formulated in combination. Lopinavir/ritonavir inhibits viral protease and seems to reduce the viral load in COVID-19 patients. However, the clinical evidence for this therapy remains limited, and several clinical trials are currently ongoing. Common side effects of lopinavir/ritonavir include gastrointestinal disturbance, insomnia, dyslipidemia, diabetes mellitus, pancreatitis, hepatic disorders, and numerous drug interactions [75].

Several authors reported the adverse effects of ritonavir on the human retina. Roe et al. first described a bilateral macular retinal pigment epitheliopathy with parafoveal telangiectasias and intraretinal crystal deposits in three HIV-positive patients on a long-term therapy with ritonavir [76]. The most common clinical findings are pigmentary changes of the macula that can present with a granular pattern, a bull's eye shape, or less specific patterns and can lead to severe vision loss. Bone spicule-like pigment changes in the midperipheral retina and crystalline intraretinal deposits can also occur. OCT features include macular thinning with outer retinal layers atrophy and loss of the ellipsoid zone, which also shows an abnormal hyperreflectivity. Ritonavir-associated retinal toxicity has been reported only with chronic use. The shortest time before diagnosis described in the literature is 19 months [76]. As for HIV cases, the suggested dosage of lopinavir/ritonavir for COVID-19 patients is 400/100 mg twice daily. In most COVID-19 cases, the duration of treatment is 5 to 7 days [75]. Therefore, a retinal toxicity caused by a short-term use of lopinavir/ritonavir seems unlikely in COVID-19 patients.

#### 3.3.3. Immunomodulatory Drugs

Interferons (IFN), a family of cytokines with antiviral properties, have been suggested as a potential treatment for COVID-19 due to their antiviral, antiproliferative, and immunomodulatory activities.

Among IFN subtypes, IFN-beta-1 may account for a safe and easy-to-upscale treatment against COVID-19 in the early stages of infection [64].

Interferon-associated retinopathy often presents with cotton-wool spots, retinal hemorrhages, and other retinal microvascular irregularities. These changes occur most notably around the optic nerve head and in the posterior pole [77]. The retinopathy typically presents 3 to 5 months after treatment begins; however, it can present as early as 2 to 6 weeks into the treatment [78]. Fortunately, the ocular findings of interferon-associated retinopathy appear to reverse with cessation of treatment.

Interleukin-1 inhibitors (e.g., anakinra) and interleukin-6 inhibitors (e.g., sarilumab, siltuximab, and tocilizumab) are also under evaluation for the treatment of COVID-19. Endogenous IL-1 and IL-6 are elevated in patients with SARS-CoV-2 infection, and they could be important mediators of severe systemic inflammatory responses in these patients. To date, no studies on the use of IL-1 and IL-6 inhibitors in patients with COVID-19 are published, although several clinical trials are underway [64].

Some studies reported an association between high dose of anakinra and nystagmus. A case report described some ocular adverse events related to tocilizumab, such as bilateral retinopathy with multifocal cotton-wool spots and retinal hemorrhages, bilateral papilledema, HTLV-1 uveitis, viral conjunctivitis, and ophthalmic herpes zoster infection [79, 80].

## 4. Conclusions

The rapid progression of the COVID-19 pandemic has created significant challenges for the public, as well as healthcare professionals around the world. Knowledge regarding virus incubation, transmission, and shedding is crucial for the reduction of new cases and protection of healthcare professionals. Patient management has surely changed, also from an ophthalmological point of view. There have been several reports of eye redness and irritation in COVID-19 patients, both anecdotal and published, suggesting that conjunctivitis may be an ocular manifestation of SARS-CoV-2 infection. As conjunctivitis is a common eye condition, ophthalmologists may be the first medical professionals to evaluate a patient with COVID-19. The real incidence of conjunctivitis in COVID-19 patients is not certain yet, and this may be the only manifestation of infection from SARS-CoV-2. Therefore, special care must be taken when examining patients with signs and symptoms of viral conjunctivitis. It is mandatory to investigate the presence of respiratory symptoms or any element that, from an epidemiological point of view, suggests a potential infection from SARS-CoV-2. Conjunctival swabs may represent a help in clinical practice, even though a negative analysis via RT-PCR does not exclude the infection.

Other patients are going to require ophthalmological attention.

Many people throughout the world are having a more sedentary lifestyle and less healthy diet, due to restrictions and economic difficulties. This may lead to poor diabetes control, and it potentially increases the risk of developing more severe forms of diabetic retinopathy. In addition, diabetic patients are receiving less strict medical controls, due to lockdown.

Implications of therapies used to treat COVID-19 patients are likely to be of ophthalmological interest too. Since a targeted therapy against SARS-CoV-2 is lacking, many drugs have been used synergically, usually at high dosage. Most of them may develop retinopathies as side effects. When the pandemic is over, ophthalmologists may be called to assess the extent of the retinal and visual damage exerted by these life-saving therapies.

Most patients requiring mechanical ventilation may experience disorders of the eye surface, with a variable degree of severity. It may be difficult to treat these occurrences while the patient remains in the ICU; however, they may lead to sight-threatening complications, like bacterial superinfection and corneal abrasions. Also, optic neuritis and acute angle-closure glaucoma are a rare complication of prone positioning, which has proven itself efficient in treating cases of severe COVID-19 pneumonia. To sum up, the health care professionals are nowadays facing an unprecedented global health issue, which is affecting each medical specialty. It is important to exert the maximum effort on reducing the contagion rate and to treat patients to the best of our abilities, despite the pandemic.

## References

[1] Guan W.-j., Ni Z.-y., Hu Y. (2020). Clinical characteristics of coronavirus disease 2019 in China. *New England Journal of Medicine*.

[2] Petersen E., Hui D., Hamer D. H. (2020). Li Wenliang, a face to the frontline healthcare worker. The first doctor to notify the emergence of the SARS-CoV-2, (COVID-19), outbreak. *International Journal of Infectious Diseases*.

[3] Lu R., Zhao X., Li J. (2020). Genomic characterisation and epidemiology of 2019 novel coronavirus: implications for virus origins and receptor binding. *The Lancet*.

[4] Gulati A., Pomeranz C., Qamar Z. (2020). A comprehensive review of manifestations of novel coronaviruses in the context of deadly COVID-19 global pandemic. *The American Journal of the Medical Sciences*.

[5] Seah I., Agrawal R. (2020). Can the coronavirus disease 2019 (COVID-19) affect the eyes? A review of coronaviruses and ocular implications in humans and animals. *Ocular Immunology and Inflammation*.

[6] Thabet L., Mhalla S., Naija H. (2020). SARS-CoV-2 infection virological diagnosis. *La Tunisie Medicale*.

[7] Lu C. W., Liu X. F., Jia Z. F. (2020). 2019-nCoV transmission through the ocular surface must not be ignored. *The Lancet*.

[8] Zhou L., Xu Z., Castiglione G. M. (2020). ACE2 and TMPRSS2 are expressed on the human ocular surface, suggesting susceptibility to SARS-CoV-2 infection. *The Ocular Surface*.

[9] Lange C., Wolf J., Auw‐Haedrich C. (2020). Expression of the COVID-19 receptor ACE2 in the human conjunctiva. *Journal of Medical Virology*.

[10] Ma D., Chen C.-B., Jhanji V. (2020). Expression of SARS-CoV-2 receptor ACE2 and TMPRSS2 in human primary conjunctival and pterygium cell lines and in mouse cornea. *Eye*.

[11] Colavita F., Lapa D., Carletti F. (2020). SARS-CoV-2 isolation from ocular secretions of a patient with COVID-19 in Italy with prolonged viral RNA detection. *Annals of Internal Medicine*.

[12] Wu P., Duan F., Luo C. (2020). Characteristics of ocular findings of patients with coronavirus disease 2019 (COVID-19) in Hubei Province, China. *JAMA Ophthalmology*.

[13] Cheema M., Aghazadeh H., Nazarali S. (2020). Keratoconjunctivitis as the initial medical presentation of the novel coronavirus disease 2019 (COVID-19). *Canadian Journal of Ophthalmology*.

[14] Navel V., Chiambaretta F., Dutheil F. (2020). Haemorrhagic conjunctivitis with pseudomembranous related to SARS-CoV-2. *American Journal of Ophthalmology Case Reports*.

[15] Seah I. Y. J., Anderson D. E., Kang A. E. Z. (2020). Assessing viral shedding and infectivity of tears in coronavirus disease 2019 (COVID-19) patients. *Ophthalmology*.

[16] Loffredo L., Pacella F., Pacella E., Tiscione G., Oliva A., Violi F. (2020). Conjunctivitis and COVID-19: a meta-analysis. *Journal of Medical Virology*.

[17] Khavandi S., Tabibzadeh E., Naderan M., Shoar S. (2020). Corona virus disease-19 (COVID-19) presenting as conjunctivitis: atypically high-risk during a pandemic. *Contact Lens & Anterior Eye*.

[18] Scalinci S. Z., Trovato battagliola E. (2020). Conjunctivitis can be the only presenting sign and symptom of COVID-19. *IDCases*.

[19] Wu P., Liang L., Chen C., Nie S. (2020). A child confirmed COVID-19 with only symptoms of conjunctivitis and eyelid dermatitis. *Graefe’s Archive for Clinical and Experimental Ophthalmology*.

[20] Chen L., Liu M., Zhang Z. (2020). Ocular manifestations of a hospitalised patient with confirmed 2019 novel coronavirus disease. *British Journal of Ophthalmology*.

[21] Korobelnik J.-F., Loewenstein A., Eldem B. (2020). Guidance for anti-VEGF intravitreal injections during the COVID-19 pandemic. *Graefe’s Archive for Clinical and Experimental Ophthalmology*.

[22] Veritti D., Sarao V., Bandello F., Lanzetta P. (2020). Infection control measures in ophthalmology during the COVID-19 outbreak: a narrative review from an early experience in Italy. *European Journal of Ophthalmology*.

[23] Verdoni L., Mazza A. (2020). An outbreak of severe Kawasaki-like disease at the Italian epicentre of the SARS-CoV-2 epidemic: an observational cohort study. *The Lancet*.

[24] Riphagen S., Gomez X., Gonzalez-Martinez C., Wilkinson N., Theocharis P. (2020). Hyperinflammatory shock in children during COVID-19 pandemic. *The Lancet*.

[25] Jones V. G., Mills M., Suarez D. (2020). COVID-19 and Kawasaki disease: novel virus and novel case. *Hospital Pediatrics*.

[26] Jacob J. L., Polomeno R. C., Chad Z., Lapointe N. (1982). Ocular manifestations of Kawasaki disease (mucocutaneous lymph node syndrome). *Canadian Journal of Ophthalmology. Journal Canadien D’ophtalmologie*.

[27] Krogh-madsen R., Thyfault J. P., Broholm C. (2010). A 2-wk reduction of ambulatory activity attenuates peripheral insulin sensitivity. *Journal of Applied Physiology*.

[28] Marinho P. M., Marcos A. A. A., Romano A. C. (2020). Retinal findings in patients with COVID-19. *Lancet*.

[29] Vavvas D. G., Sarraf D., Sadda S. R. (2020). Concerns about the interpretation of OCT and fundus findings in COVID-19 patients in recent *Lancet* publication. *Eye*.

[30] Zhang Y., Xiao M., Zhang S. (2020). Coagulopathy and antiphospholipid antibodies in patients with COVID-19. *New England Journal of Medicine*.

[31] Greenwood G. (2015). Case report of atypical hemolytic uremic syndrome with retinal arterial and venous occlusion treated with eculizumab. *International Medical Case Reports Journal*.

[32] Rasmussen K. L., Nordestgaard B. G., Nielsen S. F. (2018). Complement C3 and risk of diabetic microvascular disease: a cohort study of 95202 individuals from the general population. *Clinical Chemistry*.

[33] Strain W. D., Chaturvedi N. (2002). Review: the renin-angiotensin-aldosterone system and the eye in diabetes. *Journal of the Renin-Angiotensin-Aldosterone System*.

[34] Gavriilaki E., Brodsky R. A. (2020). Severe COVID-19 infection and thrombotic microangiopathy: success does not come easily. *British Journal of Haematology*.

[35] Acharya S., Diamond M., Anwar S., Glaser A., Tyagi P. (2020). Unique case of central retinal artery occlusion secondary to COVID-19 disease. *IDCases*.

[36] Janssen M. C., den Heijer M., Cruysberg J. R. (2005). Retinal vein occlusion: a form of venous thrombosis or a complication of atherosclerosis?. *Thrombosis and Haemostasis*.

[37] Dinkin M., Gao V., Kahan J. (2020). COVID-19 presenting with ophthalmoparesis from cranial nerve palsy. *Neurology*.

[38] Wei H., Yin H., Huang M., Guo Z. (2020). The 2019 novel coronavirus pneumonia with onset of oculomotor nerve palsy: a case study. *Journal of Neurology*.

[39] Chen N., Zhou M., Dong X. (2020). Epidemiological and clinical characteristics of 99 cases of 2019 novel coronavirus pneumonia in Wuhan, China: a descriptive study. *The Lancet*.

[40] Grasselli G., Zangrillo A., Zanella A. (2020). Baseline characteristics and outcomes of 1591 patients infected with SARS-CoV-2 admitted to ICUs of the Lombardy Region, Italy. *JAMA*.

[41] Huang C., Wang Y., Li X. (2020). Clinical features of patients infected with 2019 novel coronavirus in Wuhan, China. *The Lancet*.

[42] Saritas T. B., Bozkurt B., Simsek B. (2013). Ocular surface disorders in intensive care unit patients. *Scientific World Journal*.

[43] Mela E. K., Drimtzias E. G., Christofidou M. K., Filos K. S., Anastassiou E. D., Gartaganis S. P. (2010). Ocular surface bacterial colonisation in sedated intensive care unit patients. *Anaesthesia and Intensive Care*.

[44] Mercieca F., Suresh P., Morton A., Tullo A. (1999). Ocular surface disease in intensive care unit patients. *Eye*.

[45] Hernandez E. V., Mannis M. J. (1997). Superficial keratopathy in intensive care unit patients. *American Journal of Ophthalmology*.

[46] Herbert L. (2004). Ophthalmology in anaesthesia and intensive care. *Anaesthesia & Intensive Care Medicine*.

[47] Ghelichkhani P., Esmaeili M. (2020). Prone position in management of COVID-19 patients; a commentary. *Archives of Academic Emergency Medicine*.

[48] Guérin C., Reignier J., Richard J.-C. (2013). Prone positioning in severe acute respiratory distress syndrome. *New England Journal of Medicine*.

[49] Lee L. A. (2012). Risk factors associated with ischemic optic neuropathy after spinal fusion surgery. *Anesthesiology*.

[50] Nair P. N., White E. (2014). Care of the eye during anaesthesia and intensive care. *Anaesthesia & Intensive Care Medicine*.

[51] Abbas A., Hyman G. (2002). Macular hemorrhage secondary to increased intrathoracic pressure and difficult intubation. *JPMA. The Journal of the Pakistan Medical Association*.

[52] Panchabhai T., Bandyopadhyay D., Kapoor A., Akindipe O., Lane C., Krishnan S. (2016). Acute ischemic optic neuropathy with extended prone position ventilation in a lung transplant recipient. *International Journal of Critical Illness and Injury Science*.

[53] Ozcan M. S., Praetel C., Bhatti M. T., Gravenstein N., Mahla M. E., Seubert C. N. (2004). The effect of body inclination during prone positioning on intraocular pressure in awake volunteers: a comparison of two operating tables. *Anesthesia & Analgesia*.

[54] Cheng M. A., Todorov A., Tempelhoff R., McHugh T., Crowder C. M., Lauryssen C. (2001). The effect of prone positioning on intraocular pressure in anesthetized patients. *Anesthesiology*.

[55] Atwater B. I., Wahrenbrock E., Benumof J. L., Mazzei W. J. (2004). Pressure on the face while in the prone position: proneview versus prone positioner. *Journal of Clinical Anesthesia*.

[56] Duane T. D. (1972). Valsalva hemorrhagic retinopathy. *Transactions of the American Ophthalmological Society*.

[57] Hönemann C., Brandt L. (2015). Valsalva retinopathy. *A & A Case Reports*.

[58] Petsas A., Chapman G., Stewart R. (2017). Acute angle closure glaucoma—a potential blind spot in critical care. *Journal of the Intensive Care Society*.

[59] Singer M. S., Salim S. (2010). Bilateral acute angle-closure glaucoma as a complication of facedown spine surgery. *The Spine Journal*.

[60] Butty Z., Gopwani J., Mehta S., Margolin E. (2016). Horner’s syndrome in patients admitted to the intensive care unit that have undergone central venous catheterization: a prospective study. *Eye*.

[61] Li H., Liu L., Zhang D. (2020). SARS-CoV-2 and viral sepsis: observations and hypotheses. *The Lancet*.

[62] Dawson D. (2005). Development of a new eye care guideline for critically ill patients. *Intensive and Critical Care Nursing*.

[63] Rosenberg J. B., Eisen L. A. (2008). Eye care in the intensive care unit: narrative review and meta-analysis. *Critical Care Medicine*.

[64] COVID-19 Treatment Guidelines Panel (2020). *Coronavirus Diseases 2019 (COVID-19) Treatment Guidelines*.

[65] EUA Hydroxychloroquine Sulfate Health Care Provider Fact Sheet, https://www.fda.gov/media/136537/download

[66] EUA Chloroquine Phosphate Health Care Provider Fact Sheet, https://www.fda.gov/media/136535/download

[67] Gao J., Tian Z., Yang X. (2020). Breakthrough: chloroquine phosphate has shown apparent efficacy in treatment of COVID-19 associated pneumonia in clinical studies. *BioScience Trends*.

[68] Chen Z., Hu J., Zhang Z. (2020). Efficacy of hydroxychloroquine in patients with COVID-19: results of a randomized clinical trial.

[69] Gautret P., Lagier J.-C., Parola P. (2020). Hydroxychloroquine and azithromycin as a treatment of COVID-19: results of an open-label non-randomized clinical trial. *International Journal of Antimicrobial Agents*.

[70] Stokkermans T. J., Trichonas G. (2020). *Chloroquine and Hydroxychloroquine Toxicity*.

[71] Marmor M. F., Kellner U., Lai T. Y. Y., Melles R. B., Mieler W. F. (2016). Recommendations on screening for chloroquine and hydroxychloroquine retinopathy (2016 revision). *Ophthalmology*.

[72] Navajas E. V., Krema H., Hammoudi D. S. (2015). Retinal toxicity of high-dose hydroxychloroquine in patients with chronic graft-versus-host disease. *Canadian Journal of Ophthalmology*.

[73] Leung L.-S. B., Neal J. W., Wakelee H. A., Sequist L. V., Marmor M. F. (2015). Rapid onset of retinal toxicity from high-dose hydroxychloroquine given for cancer therapy. *American Journal of Ophthalmology*.

[74] Marmor M. F. (2020). COVID-19 and chloroquine/hydroxychloroquine: is there ophthalmological concern?. *American Journal of Ophthalmology*.

[75] Lopinavir/ritonavir: a rapid review of effectiveness in COVID-19, The Center for Evidence-Based Medicine, https://www.cebm.net/covid-19/lopinavir-ritonavir-a-rapid-review-of-the-evidence-for-effectiveness-in-treating-covid/

[76] Roe R. H., Jumper J. M., Gualino V. (2011). Retinal pigment epitheliopathy, macular telangiectasis, and intraretinal crystal deposits in HIV-positive patients receiving ritonavir. *Retina*.

[77] https://www.aifa.gov.it/documents/20142/0/lopinavir_ritonavir_02.04.2020.pdf/64b8cf03-acf1-e9fa-80fa-c6d3ecba5f7d

[78] Schulman J. A., Liang C., Kooragayala L. M., King J. (2003). Posterior segment complications in patients with hepatitis C treated with interferon and ribavirin. *Ophthalmology*.

[79] Kadayifcilar S., Boyacioglu S., Kart H., Gursoy M., Aydin P. (1999). Ocular complications with high-dose interferon alpha in chronic active hepatitis. *Eye*.

[80] Tada A., Hashida N., Tanaka T., Nishida K. (2012). Anti-interleukin-6 receptor antibody therapy-induced retinopathy in a patient with rheumatoid arthritis. *Case Rep Rheumatol*.

